# The association between hyperuricemia and betel nut chewing in Taiwanese men: a cross-sectional study

**DOI:** 10.1186/1471-2458-13-1136

**Published:** 2013-12-05

**Authors:** Tsai-Sung Tai, Chih-Cheng Hsu, Hsiang-Chu Pai, Wen-Hsin Liu, Yueh-Han Hsu

**Affiliations:** 1 Division of Endocrinology and Metabolism, Ditmanson Medical Foundation Chia-Yi Christian Hospital, Chia-Yi City 600, Taiwan; 2 Institute of Population Health Sciences, National Health Research Institutes, Zhunan, Miaoli County 350, Taiwan; 3 Department of Health Services Administration, China Medical University and Hospital, Taichung City 404, Taiwan; 4 Department of Nursing, Min-Hwei Junior College of Health Care Management, Tainan City 736, Taiwan; 5 Division of Family Medicine, Ditmanson Medical Foundation Chia-Yi Christian Hospital, Chia-Yi, Taiwan; 6 Department of Health Services Administration, China Medical University, Taichung City 404, Taiwan; 7 Division of Nephrology, Ditmanson Medical Foundation Chia-Yi Christian Hospital, 539 Chung Hsiao Road, Chia-Yi City 600, Taiwan

**Keywords:** Betel nut, Chronic kidney disease, Comorbidity, Hyperuricemia, Logistic regression analysis

## Abstract

**Background:**

Studies have associated betel nut chewing with cancers, metabolic syndrome, cardiovascular disorders, chronic kidney disease, and proteinuria. This study investigated whether hyperuricemia is associated with betel nut chewing in men who participated in a health check-up program.

**Methods:**

From hospital records, we identified a total of 11,991 men who participated in the health check-up program from 2003 to 2009. They were divided into hyperuricemic group and non-hyperuricemic group. Laboratory tests, medical history, and status of cigarette smoking, alcohol consumption, and betel nut chewing were compared between the 2 groups. We calculated odds ratio (OR) and 95% confidence interval (CI) of hyperuricemia in association with betel nut consumption and other factors.

**Results:**

Compared with the non-hyperuricemic group, the hyperuricemic group was slightly older (59.4 vs. 58.6 years) but less prevalent with betel nut use (11.8 vs. 13.6%, p = 0.003). Multivariable logistic regression analysis showed that hyperuricemia was negatively associated with betel nut chewing (OR 0.75, 95% CI 0.66-0.84), older age (OR 0.84, 95% CI 0.77-0.93), and diabetes mellitus (OR 0.57, 95% CI 0.50-0.64). On the other hand, hyperuricemia was positively associated with body mass index (OR 1.75, 95% CI 1.62-1.90), drinking (OR 1.36, 95% CI 1.25-1.49), hypertension (OR 1.41, 95% CI 1.30-1.52), mixed hyperlipidemia (OR 1.84, 95% CI 1.33-2.54), chronic kidney disease (OR 3.28, 95% CI 2.94-3.65), and proteinuria (OR 1.22, 95% CI 1.08-1.38). Smoking, hypercholesterolemia, and hypertriglyceridemia had no significant association with hyperuricemia.

**Conclusion:**

Our data suggest that betel nut chewing is negatively associated with hyperuricemia.

## Background

Betel nut chewing, a common unhealthy behavior in Southeast Asian countries, is the fourth most popular risky habit in the world after smoking, drinking alcohol, and caffeine consumption. Approximately 600 million people use this substance globally [1,2]. The prevalence rate of betel nut chewing in Taiwan has been estimated to be 12.2% – 16.5% in two large studies [3,4]. Previous studies have shown that betel nut chewing is related to several disorders, such as metabolic syndrome [5], cardiovascular disease (CVD) [6,7], hypertension [8], chronic kidney disease [9] and proteinuria [10].

Recent epidemiological and clinical studies have also suggested a close relationship between hyperuricemia and metabolic syndrome [11], CVD [12], hypertension [13], and chronic kidney disease [14]. Based on both the revised and original National Cholesterol Education Program Adult Treatment Panel (NCEP/ATP) III criteria defined metabolic syndrome, Choi et al. found that individuals with uric acid levels of ≥10 mg/dL are 3-fold more likely than those with serum uric acid levels of <6 mg/dL to be prevalent with metabolic syndrome [11]. In an Italian study, Verdecchia et al. reported that study population with a higher serum uric acid level (>6.2 mg/dL for men and >4.6 mg/dL for women) have increased risk for cardiovascular events and fatal cardiovascular events [12]. A Taiwanese population study reported that adults older than 40 with hyperuricemia had an odds ratio (OR) of 3.65 for chronic kidney disease [14].

Although betel nut chewing and hyperuricemia share a high degree of similarity in their comorbid conditions, there is no known research exploring the relationship between betel nut chewing and hyperuricemia. The association between them deserves further clarification. The purpose of this study was to investigate the association between betel nut chewing and hyperuricemia.

## Methods

### Study subjects

Detailed study design and definitions of variables for this study have been reported in a previous study [9]. Briefly, all 34 372 participants attending the Taiwan National Health Insurance Bureau sponsored health check-up program at our hospital from 2003 to 2009 were included in this study. For 1481 participants with multiple check-ups, only their first check-up records were used. Participants (n = 5409) with incomplete records were excluded. The institutional review board of the Ditmanson Medical Foundation Chia-Yi Christian Hospital has approved the present study.

### Clinical and demographic variables

All participants were required to complete a self-reported questionnaire for information on sociodemographic status, lifestyle, and personal medical history with the assistance of trained volunteers. Frequency and amount of smoking, drinking and betel nut chewing “in the past 6 months” were reported. They reported personal medical history in regarding to chronic kidney disease, diabetes mellitus, hypertension, hyperlipidemia, hyperuricemia, liver dysfunction, and anemia. The health check up consisted of anthropometric measurements, and blood and urine specimens measured with standard automated technology after 8 hours of overnight fasting, such as systolic blood pressure (SBP), diastolic blood pressure (DBP), serum creatinine, total cholesterol, triglycerides, uric acid, fasting blood glucose, alanine aminotransferase, hemoglobin, white blood cell count, and urinary biochemical and microscopic examination of sediment. The Modification of Diet in Renal Disease (MDRD) formula was used to calculate estimated glomerular filtration rate (eGFR) [15].

### Definitions of variables

Hyperuricemia was defined as a serum uric acid concentration >7.0 mg/dl for men [16]. Chronic kidney disease was defined as an eGFR less than 60 ml/min/1.73 m^2^ calculated using the MDRD formula [15]. Hypertension was defined as the use of antihypertensive medications, or SBP >140 mmHg or DBP >90 mmHg [17]. Diabetes mellitus was defined as a fasting plasma glucose level equal to or over 126 mg/dl or a known history of diabetes with or without medication [18]. Hyperlipidemia was defined as having a serum total cholesterol level >200 mg/dl, and/or triglycerides > 150 mg/dl, or a past personal history of high total cholesterol or triglycerides with or without medication [19]. Participants were considered to be non-smokers if they reported that they did not smoke, or as smokers if they smoked socially, occasionally or irregularly regardless of the amount in the previous 6 months. They were considered to be non-chewers if they reported that they did not consume any betel nut at all, or as chewers if they consumed betel nut socially, occasionally or irregularly in the previous 6 months. They were considered to be non-drinkers if they reported that they did not consume any alcohol and as drinkers if they consumed alcohol socially, occasionally or irregularly regardless of the amount in the previous 6 months. Proteinuria was defined as having a +/− or heavier protein response (including + to 4+) in a urine dipstick test.

### Laboratory measures

Biochemical tests including total cholesterol, triglycerides, uric acid, and serum creatinine were measured by an automatic analyzer (Hitachi 7170, Hitachi High Technologies Co. Tokyo, Japan). The reagents for all biochemical tests were manufactured by Wako Pure Chemical Industries, Ltd., Japan. Dipstick urine analysis was performed by an automated chemical analyzer (URISYS 2400, Roche Diagnostic, Germany).

### Statistical analysis

Comparative data about the presence or absence of hyperuricemia were reported as means and standard deviations or numbers and percentages as appropriate. Demographics, clinical characteristics and comorbid conditions were analyzed by the Student’s *t* test for continuous variables and by the *x*^2^ test for categorical variables.

To determine the association between betel nut chewing and hyperuricemia, we utilized a univariate and stepwise multivariate logistic regression models. Covariates significant at the univariate analysis were included in the multivariate analysis to further clarify associations between betel nut chewing and hyperuricemia and other variables. In addition to examining confounding effects between betel nut chewing and other variables, we checked evidence of interaction among them as needed.

All analyses were carried out using the SPSS for Windows statistical software package version 18 (SPSS Inc., Chicago, IL, USA). For all difference estimates and odds ratios, we calculated 95% confidence intervals (95% CIs). *P* values < 0.05 were considered to be statistically significant.

## Results

A total of 11,991 male participants (mean age 58.9 ± 12.1 years) were included in the data analysis (Table 1). Among them, 4,702 (39.2%) individuals were found to have hyperuricemia with the mean level of 8.36 (SD 1.17) mg/dl. Subjects with hyperuricemia tended to be older and to have higher mean values of body mass index (BMI), SBP, DBP, serum creatinine, total cholesterol, and triglycerides, but lower values of eGFR and fasting plasma glucose (p <0.001 for all).

**Table 1:** Demographic and biochemical variables compared between hyperuricemic and non-hyperuricemic groups

	**Over all**	**Hyperuricemia (−)**	**Hyperuricemia (+)**	
	**(n = 11991)**	**(n = 7289, % = 60.8)**	**(n = 4702, % = 39.2)**	**p-value**
Age (years)	58.9 ± 12.1	58.6 ± 11.9	59.4 ± 11.3	<0.001*
eGFR (ml/min/1.73 m^2^)	73.9 ± 16.8	77.4 ± 15.7	68.5 ± 17.2	<0.001*
BMI (Kg/m^2^)	25.11 ± 3.47	24.7 ± 3.42	25.8 ± 3.43	<0.001*
Systolic BP (mmHg)	133.1 ± 19.7	131.5 ± 19.3	135.6 ± 19.9	<0.001*
DiastolicBP (mmHg)	79.3 ± 12.3	78.1 ± 11.9	81.0 ± 12.6	<0.001*
Serum creatinine (mg/dL)	1.15 ± 0.35	1.09 ± 0.27	1.24 ± 0.43	<0.001*
Serum total cholesterol (mg/dL)	206.5 ± 41.3	204.8 ± 40.4	209.2 ± 42.6	<0.001*
Serum triglyceride (mg/dL)	156.7 ± 166.1	143.0 ± 141.1	177.8 ± 196.8	<0.001*
Serum uric acid (mg/dL)	6.76 ± 1.64	5.74 ± 0.92	8.36 ± 1.17	<0.001*
Fasting plasma glucose (mg/dL)	107.1 ± 40.5	109.5 ± 45.6	103.3 ± 30.6	<0.001*

*p-value < 0.001.

Results are expressed as means ± standard deviation (SD).

Table 2 shows that the prevalence of betel nut chewing was lower in participants with hyperuricemia than in participants without hyperuricemia (11.8% vs. 13.6%). Those with hyperuricemia were more prevalent with alcohol consumption, chronic kidney disease, hypertension, mixed hyperlipidemia, and proteinuria, but less likely to be overweight and to have diabetes. The logistic regression analysis measured ORs for the corresponding associations are shown in Table 3.

**Table 2:** Personal behaviors and co-morbid conditions compared between hyperuricemic and non-hyperuricemic groups

	**Over all**	**Hyperuricemia (−)**	**Hyperuricemia (+)**	
	**(n = 11991)**	**(n = 7289, % = 60.8)**	**(n = 4702, % = 39.2)**	**p-value**
Smoker	4448(37.1%)	2725(37.4%)	1723(36.6%)	0.412
Drinker	3892(32.5%)	2251(30.9%)	1641(34.9%)	<0.001**
Betel Nut Chewer	1545(12.9%)	992(13.6%)	553(11.8%)	0.003*
Betel nut non-chewer	10446(87.1%)	6279(86.4%)	4149(88.2%)	0.003*
CKD	2165(18.1%)	832(11.4%)	1333(28.3%)	<0.001**
Overweight	6003(50.1%)	2777(59.1%)	3226(44.3%)	<0.001**
Diabetes	1794(15.0%)	1211(16.6%)	583(12.4%)	<0.001**
Hypertension	5437(45.3%)	2945(40.4%)	2492(53.0%)	<0.001**
Hypercholesterolemia	114(1.0%)	74(1.0%)	40(0.9%)	0.365
Hypertriglyceridemia	85(0.7%)	49(0.7%)	36(0.8%)	0.552
Mixed hyperlipidemia	168(1.4%)	72(1.0%)	96(2.0%)	<0.001**
Proteinuria	1381(11.5%)	724(9.9%)	657(14.0%)	<0.001**

Results are expressed as n (%).

overweight denotes BMI > = 25.

*p-value < 0.01; **p-value < 0.001.

**Table 3:** The crude and adjusted odds ratios (aOR) for hyperuricemia by logistic regression analysis

	**Crude OR**	**95% CI**	**aOR**	**95% CI**
Age				
≥60 years old	1.12	(1.04-1.20)*	0.84	(0.77-0.92)**
<60 years old	1.00		1.00	
BMI				
≥25	1.82	(1.69-1.96)**	1.75	(1.62-1.90)**
<25	1.00		1.00	
Betel nut chewing				
(+)	0.85	(0.76-0.95)*	0.75	(0.66-0.84)**
(−)	1.00		1.00	
Smoking				
(+)	0.97	(0.90-1.05)	1.02	(0.93-1.10)
(−)	1.00		1.00	
Alcohol intake				
(+)	1.20	(1.11-1.30)**	1.36	(1.25-1.49)**
(−)	1.00		1.00	
Diabetes				
(+)	0.71	(0.64-0.79)**	0.57	(0.50-0.64)**
(−)	1.00		1.00	
Hypertension				
(+)	1.66	(1.55-1.79)**	1.41	(1.30-1.52)**
(−)	1.00		1.00	
Hypercholesterolemia				
(+)	0.84	(0.57-1.23)	0.83	(0.55-1.24)
(−)	1.00		1.00	
Hypertriglyceridemia				
(+)	1.14	(0.74-1.76)	1.09	(0.70-1.72)
(−)	1.00		1.00	
Mixed hyperlipidemia				
(+)	2.09	(1.54-2.84)**	1.84	(1.33-2.54)**
(−)	1.00		1.00	
Chronic kidney disease				
(+)	3.07	(2.79-3.38)**	3.28	(2.94-3.65)**
(−)	1.00		1.00	
Proteinuria				
(+)	1.47	(1.32-1.65)**	1.22	(1.08-1.38)*
(−)	1.00		1.00	

*p-value < 0.01; **p-value < 0.001.

*a:* The adjusted odds ratios were measured controlling for age, BMI, betel nut chewing, smoking, alcohol intake, diabetes, hypertension, hypercholesterolemia, hypertriglyceridemia, mixed hyperlipidemia, chronic kidney disease and proteinuria.

The multivariate logistic regression model showed that subjects with hyperuricemia had an OR of 0.75 (95% confidence interval (CI) 0.66-0.84) in association with betel nut chewing (Table 3). Older men and men with diabetes were also less likely to be hyperuricemia. Higher BMI, drinking, hypertension hyperlipidemia, chronic kidney disease and proteinuria were factors associated with hyperuricemia. Chronic kidney disease had the strongest association with hyperuricemia (adjusted OR 3.28, 95% CI 2.94-3.65).

Table 4 depicts the association between betel nut chewing and hyperuricemia by stratified levels of age and BMI, and subjects with and without diabetes and hypertension. The association was significant for subjects of younger age, higher BMI, no diabetes and no hypertension.

**Table 4:** Odds ratio of hyperuricemia associated with betel nut chewing by age, body mass index, diabetes and hypertension

	**Odds ratio**	**95% CI**	**P value**
Overall *a*	0.745	(0.658-0.844)	<0.001***
Age *b*			
≥60 years old	0.879	(0.697-1.109)	0.277
<60 years old	0.705	(0.607-0.818)	<0.001***
BMI *b*			
≥25	0.670	(0.571-0.786)	<0.001***
<25	0.874	(0.718-1.065)	0.182
Diabetes *b*			
(+)	0.796	(0.579-1.094)	0.160
(−)	0.833	(0.731-0.95)	0.006**
Hypertension *b*			
(+)	0.799	(0.661-0.967)	0.021*
(−)	0.705	(0.597-0.833)	<0.001***

*p-value < 0.05; **p-value < 0.01; ***p-value < 0.001.

*a*: Multivariate logistic regression analysis conducted with all adjustment factors, including age, body mass index (BMI), smoking, alcohol intake, hypertension, diabetes, chronic kidney disease, hypercholesterolemia, hypertriglyceridemia, mixed-hyperlipidemia, proteinuria.

*b*: Multivariate logistic regression analysis conducted with the rest of the variables, in addition to specific variables under stratified conditions as presence and absence.

## Discussion

In this cross-sectional study, we found that hyperuricemia is positively associated with BMI, drinking, hypertension, chronic kidney disease, and proteinuria, but negatively associated with old age (≧60 y/o), betel nut chewing, and diabetes mellitus.

To the best of our knowledge, this study was the first to investigate the association between betel nut chewing and hyperuricemia. Several mechanisms may provide potential explanations why betel nut chewers have a lower prevalence of hyperuricemia. The most important chemical components of betel nut are alkaloids [20]. Kanbara and Seyama [21] found a lower serum uric acid level with an alkaline diet than with an acidic diet. Uric acid is more actively reabsorbed with acidic urine than with alkaline urine. They also found that alkalization of urine by eating alkaloid food was an effective way to excrete uric acid via the urine [22]. Further research on uric acid excretion for betel nut chewers is warranted.

Our data showed that the relationship between hyperuricemia and diabetes mellitus is consistent with previous studies [23-26]. A possible mechanism for this inverse association may be related to the inhibition of uric acid reabsorption in the proximal tubules by high glucose levels [27,28].

A recent research carried out with 14,362 Taiwanese showed that, for men, values of uric acid decreased as age increased [29]. The finding is consistent with our result. Another longitudinal study for men showed that the incidence of hyperuricemia increased in parallel with the rise in BMI, with a correlation coefficient of 0.282 (p <0.0001) between serum uric acid levels and BMI [30]. The results also support our finding of the positive association between hyperuricemia and BMI.

Our data also showed that drinking alcohol and hypertension were positively associated with hyperuricemia. A recent meta-analysis of 18 published prospective cohort studies with 55,607 subjects showed that hyperuricemia was associated with an increased risk for incidental hypertension (adjusted risk ratio 1.41, 95% CI 1.23-1.58) [31]. The overall risk for incidental hypertension increases by 13% per 1 mg/dl increase in serum uric acid level. Several possible mechanisms for hyperuricemia in the development of hypertension have been proposed. Higher uric acid levels could lead to endothelial cell dysfunction via nitric oxide synthetase [32,33] and stimulate vascular smooth muscle cell proliferation [34]. Uric acid may directly stimulate the renin-angiotensin system [35,36] and cause renal afferent arteriolopathy and tubulointerstitial nephritis, leading to higher blood pressure [36]. Renal lesions and hypertension are prevented by lowering uric acid levels with a xanthine oxidase inhibitor or a uricosuric agent and reversed by an angiotensin-converting enzyme inhibitor [37].

Previous studies support our findings in associations between hyperuricemia and mixed hyperlipidemia, chronic kidney disease and proteinuria [38-43]. Uric acid can induce pre-glomerular arterial disease, vascular proliferation, renal inflammation, and hypertension via an activation of renin-angiotensin system and cyclooxygenase-2, which in turn aggravates renal disease, endothelial dysfunction, hypertension, and cardiovascular disease [44-47].

The stratified analyses shown in Table 4 support our main outcome. Irrespective of the status in overweight, diabetes and age, the odds ratios were still all less than one. In fact, according to Table 3, co-variables with age ≧ 60, BMI < 25 or presence of diabetes were already factors for lower hyperuricemia odds, which might thus keep their ORs less than one but fail to sustain statistical significance in the stratified analyses.

There are several limitations to this study. First, this study was a cross-sectional study and causality cannot be concluded. Further prospective cohort studies are necessary. Second, information about exposure to betel nut chewing was from self-reported questionnaires, and obtained from a fixed questionnaire, which included no status of ex-uses of betel nut, alcohol drinking and smoking. Potential misclassification of that exposure is possible without information on ex-uses of these substances. More detailed histories about the amount of betel nut chewed, duration, and previous abstinence are needed. Third, the participants who took the initiative to undergo the health check-up program in this hospital may not have represented the general population; they might have had higher socio-economic status or level of education, and might have been more alert to the status of their own health. These factors may have been sources of selection bias. However, the number of participants was large enough to enable us to examine associations between betel nut chewing and hyperuricemia after multivariate logistic regression modeling with comprehensive adjustments for confounders. The prevalence of betel nut chewing in our study was also consistent with two previous large studies in Taiwan [3,4].

## Conclusions

Hyperuricemia has an inverse association with betel nut chewing, diabetes, and old age, while it has a positive association with obesity, alcohol consumption, hypertension, CKD, and proteinuria.

## Competing interests

The authors declare that they have no competing interests.

## Authors’ contributions

TST and YHH designed the study and drafted the manuscript. CCH guided the statistical works and revised the manuscripts. HCP contributed to the interpretation of data and providing comments on the draft. WHL provided administrative support and participated in the study design. All authors read and approved the final manuscript.

## Pre-publication history

The pre-publication history for this paper can be accessed here:

http://www.biomedcentral.com/1471-2458/13/1136/prepub
